# The role of G protein gene *GNB3 C825T *Polymorphism in HIV-1 acquisition, progression and immune activation

**DOI:** 10.1186/1742-4690-9-1

**Published:** 2012-01-03

**Authors:** Jennifer Juno, Jeffrey Tuff, Robert Choi, Catherine Card, Joshua Kimani, Charles Wachihi, Sandra Koesters-Kiazyk, T Blake Ball, Carey Farquhar, Francis A Plummer, Grace John-Stewart, Ma Luo, Keith R Fowke

**Affiliations:** 1 Department of Medical Microbiology, University of Manitoba, Winnipeg, Canada; 2 National Microbiology Laboratory, Public Health Agency of Canada, Winnipeg, Canada; 3 Department of Medicine, University of Washington, Seattle USA; 4 Kenya AIDS Control Program, Nairobi, Kenya; 5 Department of Medical Microbiology, University of Nairobi, Nairobi Kenya; 6 Department of Immunology, University of Manitoba, Winnipeg, Canada; 7 Department of Medicine, University of Washington, Seattle, USA; 8 Department of Epidemiology, University of Washington, Seattle, USA; 9 Department Global Health, University of Washington, Seattle USA; 10 Departments of Community Health Sciences University of Manitoba, Winnipeg Canada

**Keywords:** GNB3, HIV progression, G protein, HIV acquisition, Immune Activation

## Abstract

**Background:**

The *GNB3 C825T *polymorphism is associated with increased G protein-mediated signal transduction, SDF-1α-mediated lymphocyte chemotaxis, accelerated HIV-1 progression, and altered responses to antiretroviral therapy among Caucasian subjects. The *GNB3 *825T allele is highly prevalent in African populations, and as such any impact on HIV-1 acquisition or progression rates could have a dramatic impact. This study examines the association of the 825T polymorphism with HIV-1 acquisition, disease progression and immune activation in two African cohorts. *GNB3 *825 genotyping was performed for enrolees in both a commercial sex worker cohort and a perinatal HIV transmission (PHT) cohort in Nairobi, Kenya. *Ex vivo *immune activation was quantified by flow cytometry, and plasma chemokine levels were assessed by cytokine bead array.

**Results:**

*GNB3 *genotype was not associated with sexual or vertical HIV-1 acquisition within these cohorts. Within the Pumwani cohort, *GNB3 *genotype did not affect HIV-1 disease progression among seroconverters or among HIV-1-positive individuals after adjustment for baseline CD4 count. Maternal CD4 decline and viral load increase in the PHT cohort did not differ between genotypes. Multi-parametric flow cytometry assessment of T cell activation (CD69, HLA-DR, CD38) and Treg frequency (CD25^+^FOXP3^+^) found no differences between genotype groups. Plasma SDF-1α, MIP-1β and TRAIL levels quantified by cytokine bead array were also similar between groups.

**Conclusions:**

In contrast to previous reports, we were unable to provide evidence to suggest that the *GNB3 C825T *polymorphism affects HIV-1 acquisition or disease progression within African populations. *Ex vivo *immune activation and plasma chemokine levels were similarly unaffected by *GNB3 *genotype in both HIV-1-negative and HIV-1-positive individuals. The paucity of studies investigating the impact of *GNB3 *polymorphism among African populations and the lack of mechanistic studies make it difficult to assess the true biological significance of this polymorphism in HIV-1 infection.

## Background

Previously regarded as dispensable in HIV-1 pathogenesis, the G protein-mediated signaling cascades initiated by chemokine receptors including CCR5 and CXCR4 are now being shown to play important roles in HIV entry, viral latency and disease progression [1-3]. The gp120-co-receptor interaction activates unique signal transduction events that remove blocks to replication, and signaling through other chemokine receptors (including CCR6 and CCR7) during HIV-1 infection may also influence pathogenesis [2,3]. Induction of lymphocyte chemotaxis to sites of viral replication may promote immune activation, which is known to be a driver of HIV disease progression [3,4]. The majority of signaling events initiated by chemokine receptors are mediated by heterotrimeric G proteins comprised of an alpha, beta and gamma subunit, each of which is expressed in multiple isoforms. One beta subunit-encoding gene, guanine nucleotide binding protein beta polypeptide 3 (*GNB3*), harbours a single nucleotide polymorphism (SNP) at position 825 (rs5443) that has been widely associated with a number of health outcomes, including predisposition to diabetes, obesity, hypertension and atherosclerosis [5] (reviewed in [6]). Compared to other chronic diseases, few studies have examined the impact of the *GNB3 *SNP on immune function and susceptibility to infectious diseases, particularly HIV. Intriguingly, however, one report suggests that *GNB3 *825TT genotype is associated with accelerated HIV-1 disease progression in a Caucasian cohort, and the same group also reported improved virologic responses to HAART among HIV-1-positive 825TT patients compared to CC and CT genotypes [7,8]. To date, no data are available to support or oppose these associations in additional cohorts, and it is still unknown whether *GNB3 *genotype also influences susceptibility to HIV-1 infection.

Within African populations, where the worldwide HIV burden is highest, an effect of *GNB3 *825 genotype on HIV-1 disease progression and treatment outcome could have a dramatic impact, as the 825T allele frequency is high (80%) compared to Caucasian populations (~20%) [9]. Despite the extensive number of publications describing associations between *GNB3 *and diseases in Caucasian and Oriental populations, reviews and meta analyses highlight the difficulty of drawing firm conclusions from small sample sizes [10-12] and also emphasize the population-specific nature of many of these associations and the paucity of *GNB3 *epidemiological studies in African populations [10-14]. In many cases, genetic association studies have not been followed up with functional experiments designed to directly assess the impact of the *GNB3 C825T *SNP on health outcomes.

The impact of the *GNB3 825 *SNP on both HIV-1 acquisition and disease progression within African populations is also of particular interest in light of our recent report that a SNP in the *CD4 *gene (*CD4 C868T*, rs28919570), located in close proximity to *GNB3 *on chromosome 12, is associated with increased risk of HIV-1 acquisition in a high-risk Kenyan cohort and a mother-to-child HIV-1 transmission cohort [15,16]. Despite functional data indicating that the amino acid change caused by the 868T substitution results in altered CD4-gp120 affinity, it could not be ruled out that linkage with the *GNB3 *SNP was contributing to the observed epidemiological association between *CD4 868T *and HIV-1 acquisition.

Although the precise mechanism of action of the *GNB3 *SNP remains unclear, it is generally accepted that the presence of the 825T allele results in increased G protein-mediated signaling in numerous cell types, including immune cells [5,17]. Notably, several studies have indicated that the 825T allele can mediate increased chemotaxis of human T cells [18], and in healthy Caucasians the allele is also associated with increased CD4 T cell counts, proliferative responses to recall antigens and decreased lymphocyte apoptosis [14,19,20]. This has led some to suggest that the 825T allele is predictive of immunocompetence [19,20]. Together, these data suggest intriguing ways in which the 825TT genotype may affect HIV-1 acquisition and disease progression, either by altering signaling pathways required for HIV replication and/or through modulation of immune activation and antigen-specific cellular immune responses.

Given the reports linking *GNB3 825T *to accelerated disease progression in Caucasians, the evidence demonstrating the role of G protein signaling in establishing HIV infection and the possibility of linkage disequilibrium between the *CD4 *and *GNB3 *SNPs, we sought to characterize the impact of the *GNB3 *SNP in two African cohorts: a high-risk commercial sex worker (CSW) cohort and a low-risk perinatal HIV transmission (PHT) cohort, both located in Nairobi, Kenya. We also assessed the impact of the *GNB3 *genotype on T cell activation in both healthy and HIV-1-positive patients as well as plasma levels of the CXCR4 ligand SDF-1α, CCR5 ligand MIP-1β and apoptosis-inducing protein, TRAIL.

## Results

### Linkage disequilibrium between *CD4 868T *and *GNB3 825T*

The *GNB3 C825T *SNP was found at a frequency of 79.9% in 1031 members of the CSW cohort and 68.9% among 395 mothers in the PHT cohort, consistent with available African population data [9]. In both HIV-1-negative and HIV-1-positive groups, the *GNB3 825 *genotypes of the CSW cohort were in Hardy-Weinberg equilibrium (Table 1). Analysis of linkage disequilibrium in the CSW cohort using a LOD score cutoff threshold of 2.0 found that *GNB3 825T *was not significantly linked to *CD4 868T *(LOD = 1.14, D' = 0.338, r^2 ^= 0.0060).

**Table 1:** *GNB3 C825T *genotype frequency for HIV-1 negative and HIV-1 positive individuals in the CSW cohort

GNB3 825 Genotype	HIV-1 Negative (n = 349)	HIV-1 Positive (n = 682)
**CC (homozygous wildtype)**	15 (4.3%)	27 (4.0%)

**CT (heterozygous)**	124 (35.5%)	206 (30.2%)

**TT (homozygous variant)**	210 (60.2%)	449 (65.8%)

**CT Frequency**	0.355	0.302

**P for HWE**	0.904	0.942

**C allele** **T allele**	0.22 0.78	0.19 0.81

Note: Data are no. (%) of subjects, unless otherwise indicated. For the comparison between HIV-1-negative and HIV-1-positive subjects, p = 0.19 (chi squared test). HWE, Hardy-Weinberg equilibrium

### Association of *GNB3 825T *with HIV-1 acquisition and transmission

**Figure 1:**
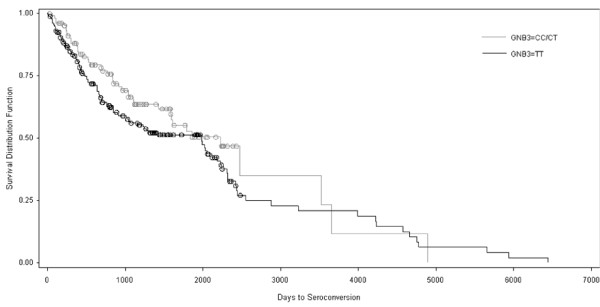
**Kaplan-Meier survival analysis of time from enrolment to seroconversion in Pumwani cohort participants**. 277 individuals with a known date of seroconversion were included in this analysis: 99 *GNB3 825CC/CT *and 178 *GNB3 825TT*. There was no difference in time to seroconversion as determined by Cox proportional hazard regression [Hazard Ratio (HR) 1.313, 95% CI 0.910, 1.895, p = 0.15]. Icons between drops in the lines represent the end of an individual's data set (censoring event).

No epidemiological analysis of *GNB3 825T *and risk of HIV-1 seroconversion has been previously reported. Survival analysis of the time from enrolment to seroconversion among participants of the CSW cohort did not significantly differ between CC/CT and TT genotypes [Cox proportional hazard analysis, Hazard Ratio (HR) 1.313, 95% CI 0.910, 1.895, p = 0.15] (Figure 1). Inclusion of the *CD4 868 *genotype in the seroconversion hazard model did not demonstrate any significant interaction between *GNB3 *and *CD4 *genotype. Consistent with these data, there were no differences in *GNB3 *genotype frequency between HIV-1-negative and HIV-1-positive CSW participants [p = 0.19, chi squared] (Table 1).

We also analysed *GNB3 825 *genotype with respect to maternal HIV-1 transmission rates either *in utero*, peripartum or via breastmilk in a low-risk Kenyan perinatal HIV transmission cohort (PHT) for which both viral load and CD4 count data were available. The overall risk of mother-to-child transmission was not associated with infant *GNB3 *genotype in an unadjusted model [HR 1.23, 95% CI: 0.76, 1.98, p = 0.40] or following adjustment for maternal viral load [p = 0.51]. Subgroup analysis of *in utero*, peripartum and breastmilk transmission events revealed no effect of 825 genotype on *in utero *transmission [Odds ratio(OR) 0.89, 95% CI: 0.38, 2.07, p = 0.78 unadjusted; OR 1.00, 95% CI 0.41, 2.44, p = 1.00 adjusted for maternal viral load] or peripartum transmission [OR 0.94, 95% CI 0.47, 1.87, p = 0.85 unadjusted; OR 0.84, 95% CI 0.40, 1.73, p = 0.63 adjusted], but did show a trend toward an increased risk of breastmilk transmission in the TT genotype group [OR 8.35, 95% CI 1.09, 64.13, p = 0.04 unadjusted; OR 7.41, 95% CI 0.94, 58.2, p = 0.06 adjusted]. This was not accompanied by a significant difference in maternal breastmilk viral load either between CC/CT and TT infants or between CC/CT and TT mothers [p = 0.54] (data not shown).

### Association of *GNB3 825T *with HIV-1 Disease progression

**Figure 2:**
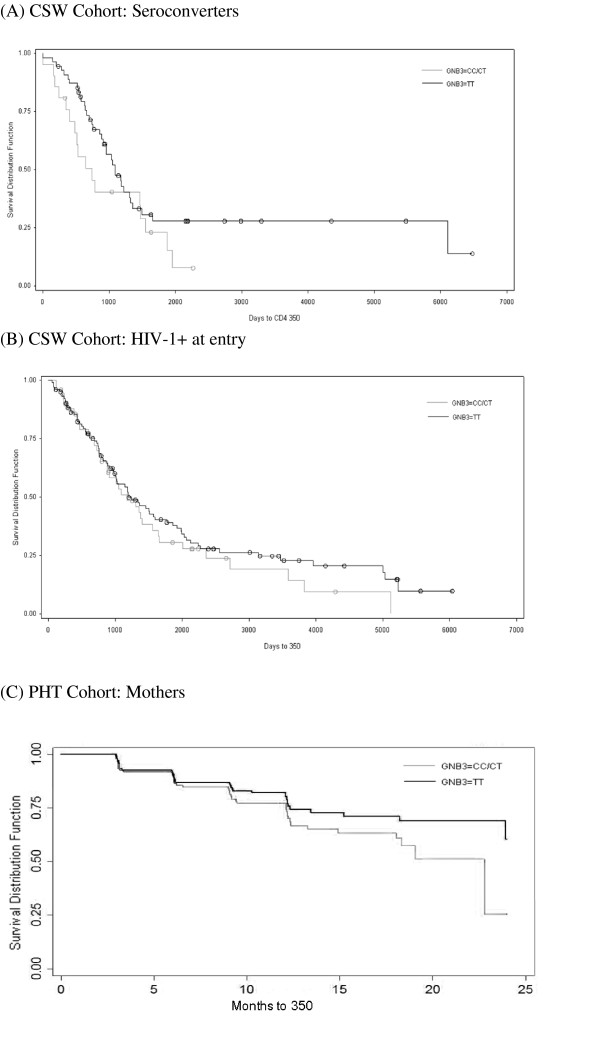
**Kaplan-Meier survival analysis of HIV disease progression to CD4 count < 350 cells/uL among**. **(A) **73 CSW cohort participants who seroconverted during follow-up: 21 *GNB3 825CC/CT *subjects and 52 *GNB3 825TT *subjects. There were no significant differences in time to CD4 < 350 as determined by Cox proportional hazard analysis [HR 0.665, 95% CI 0.369, 1.198, p = 0.1742]. **(B) **146 HIV-1-positive CSW cohort subjects: 48 *GNB3 825CC/CT *subjects and 98 *GNB3 825TT *subjects. Following adjustment for baseline CD4 count, there were no significant differences in time to CD4 < 350 as determined by Cox proportional hazard analysis [HR = 0.956, 95% CI 0.619, 1.477, p = 0.8397]. **(C) **262 PHT cohort HIV-1-positive mothers: 125 *GNB3 825CC/CT *subjects and 137 *GNB3 825TT *subjects. Following adjustment for baseline CD4 count, there were no significant differences in time to CD4 < 350 as determined by Cox proportional hazard analysis [HR = 0.75, 95% CI 0.47, 1.19, p = 0.22].

Given that Caucasian HIV-1-positive patients with the *GNB3 825TT *genotype were reported to exhibit accelerated disease progression and to respond more favourably to HAART than CC or CT genotypes [7,8], we assessed HIV disease progression in both the CSW and MCH cohorts. Within the CSW cohort, 73 genotyped participants seroconverted after their enrolment and returned for at least one follow-up visit. Among those participants, survival analysis of time to CD4 counts of < 350 cells/uL did not show any significant effect of the *GNB3 825 *genotype, nor was there any interaction with the *CD4 868 *genotype [HR 0.665, 95% CI 0.369, 1.198, p = 0.1742] (Figure 2a). Given the relatively small sample size of seroconverter patients, we also analysed disease progression among 146 women who were HIV+ with CD4 counts > 500 at the time of recruitment. Because the seroconversion dates for these women were not known, the time to CD4 < 350 cells/uL and CD4 < 250 cells/uL was adjusted for baseline CD4 count. After adjustment, there was no significant difference in time to progression in 825 CC/CT patients compared to 825 TT patients, nor was there any interaction with *CD4 *genotype [CD4 < 350: HR = 0.956, 95% CI 0.619, 1.477, p = 0.8397; CD4 < 250: HR = 0.966, 95% CI 0.594, 1.570, p = 0.8879] (Figure 2b).

Although infant HIV-1 progression data were not available for the transmission events in the PHT cohort, maternal data regarding progression to CD4 < 350 cells/uL, monthly rate of CD4 decline, death and viral load were available. CD4 decline to < 350 cells/uL did not significantly differ between 825TT and 825CC/CT genotypes before or after adjustment for baseline CD4 count [HR = 0.75, 95% CI 0.47, 1.19, p = 0.22 adjusted] (Figure 2c). Overall risk of death was not affected by GNB3 genotype [HR = 1.94, 95% CI 0.46, 8.18, p = 0.36]. Linear mixed modeling analysis of CD4 loss over two years of follow-up demonstrated a trend toward slower CD4 decline among 825TT genotype mothers, although the difference was only an average of 2.82 cells per month (9.46 cells/month among 825 CC/CT versus 6.64 cells/month among 825TT) [p = 0.08]. Similar modeling of viral load change over time did not reveal any significant effect of GNB3 genotype on viral load increase [p = 0.5] (not shown).

### Differences in Immune Activation Associated with GNB3 Genotype

**Figure 3:**
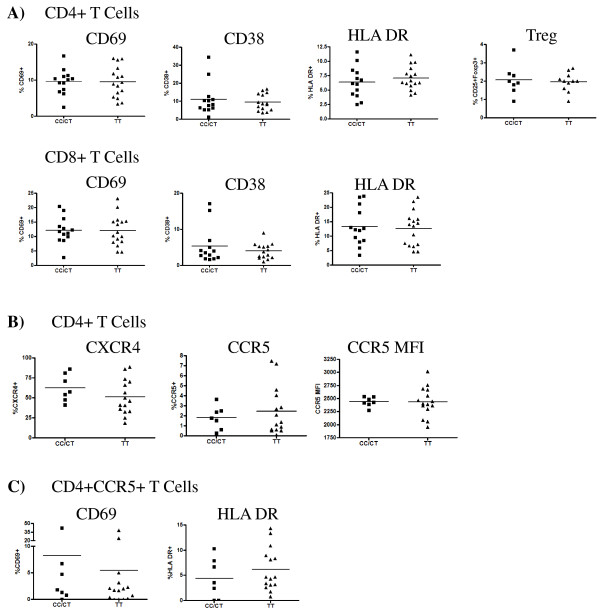
**Expression of *ex vivo *cell surface markers measured by flow cytometry among HIV-1-negative subjects**. **(A) **Expression of CD69, HLA-DR, CD38 and regulatory T cells (Tregs; defined as CD3+CD4+CD25+FOXP3+) among HIV-1-negative CSW cohort subjects. Values are expressed as a percentage of the parental CD4+ or CD8+ T cell population, as indicated. CD38 and HLA-DR plots are representative of two distinct studies. There were no significant differences in expression levels between *GNB3 825CC/CT *and *825TT *groups as measured by Mann-Whitney test [p > 0.1 for all]. **(B) **Expression of HIV-1 co-receptors CCR5 and CXCR4 expressed as a percentage of the parental CD4+ T cell population. There were no significant differences in expression levels or CCR5 median fluorescence intensity (MFI) between *GNB3 825CC/CT *and *825TT *groups as measured by Mann-Whitney test [p > 0.1 for all]. **(C) **Expression of activation markers CD69 and HLA DR on CD4+CCR5+ T cells. There were no significant differences in expression levels between *GNB3 825CC/CT *and *825TT *groups as measured by Mann-Whitney test [p > 0.1 for all].

The potential for *GNB3 *genotype to influence lymphocyte chemotaxis, cellular activation, apoptotic pathways and CD4 counts [18] could have important implications for HIV-1 disease progression, which can be driven by increasing immune activation and apoptosis [4]. The only data available describing T cell immune activation in subjects with varying *GNB3 *genotypes is reported in an assessment of healthy Caucasian individuals [20]. No differences in HLA DR expression on T cells were observed, and although bulk CD4 counts were shown to be increased in Caucasian subjects, the study did not measure any CD4+ T cell subsets such as regulatory T cells (Tregs). Therefore, to determine the effect in a genetically distinct population we assessed the *ex vivo *expression of CD69, HLA-DR and CD38, and the proportion of Tregs in both healthy and HIV-1-positive women from the Kenyan CSW cohort. In healthy women, no differences in expression of CD69 (acute activation), HLA-DR or CD38 (chronic activation) were observed between CC/CT and TT genotypes on either CD4+ or CD8+ T cells [Mann-Whitney test, p > 0.1 for all]. Expression patterns of HLA-DR and CD38 were confirmed to be similar in a second cross-sectional study [Mann-Whitney test, p > 0.1 for all] (Figure 3a). We also found no differences in Treg frequency, expressed as a percentage of CD4+ T cells [Mann-Whitney test, p = 0.96] (Figure 3a). Although activated CD4+ T cells are generally considered to be prime targets for HIV-1 infection and replication, cellular susceptibility to HIV-1 infection requires the expression of either the CCR5 (in early infection) or CXCR4 (in late infection) co-receptor. To more specifically characterize T cell susceptibility to infection, we compared the percentage of cells expressing either CXCR4 or CCR5 between *GNB3 *genotypes, but found no differences between 825 CC/CT and TT groups [Mann-Whitney test, p > 0.1 for both] (Figure 3b). As most infecting viruses utilize the CCR5 co-receptor, we also compared the density of CCR5 expression on CD4+ T cells (as measured by median fluorescence intensity) (Figure 3b) and the activation state of CD4+CCR5+ cells (as measured by CD69 and HLA DR expression) (Figure 3c). No differences in any of these parameters were observed between genotype groups [Mann-Whitney test, p > 0.1 for all].

**Figure 4:**
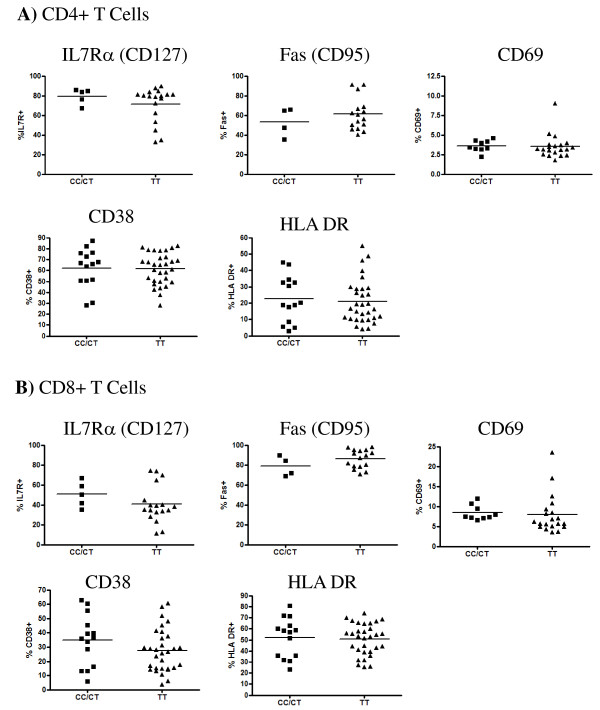
**Expression of *ex vivo *cell surface markers among HIV-1-positive subjects**. Expression of IL-7Rα, Fas, CD69, HLA-DR, and CD38 was among HIV-1-positive CSW cohort participants. Values are expressed as a percentage of the parental **(A) **CD4+ or **(B) **CD8+ T cell population, as indicated. CD69 plots are representative of two distinct studies, CD38 plots represent three distinct studies and HLA-DR plots represent four distinct studies. There were no significant differences in expression levels between *GNB3 825CC/CT *and *825TT *groups as measured by Mann-Whitney test [p > 0.1 for all].

In HIV+ women, expression of CD69, HLA-DR and CD38 did not differ between *GNB3 *genotype groups on either CD4+ or CD8+ T cells, before or after adjustment for CD4 count [Mann-Whitney test, p > 0.1 for all; ANCOVA with CD4 count as covariate, p > 0.1 for all] (Figure 4). Comparison of HLA-DR expression between genotypes was replicated in 4 cross-sectional studies, and CD38 expression was assessed in 3 replicate studies. Additionally, we measured expression of IL-7Rα (CD127) and Fas (CD95), both known correlates of HIV-1 disease progression [21,22]. Expression of both markers did not differ between GNB3 genotypes, either before or after adjustment for CD4 count [Mann-Whitney and ANCOVA, p > 0.1 for all] (Figure 4). Finally, the proportion of Tregs expressed as a percentage of CD3+ cells was also similar between genotype groups [Mann-Whitney test, p = 0.91] (not shown).

### Plasma SDF-1α, MIP-1β and TRAIL Quantification

**Figure 5:**
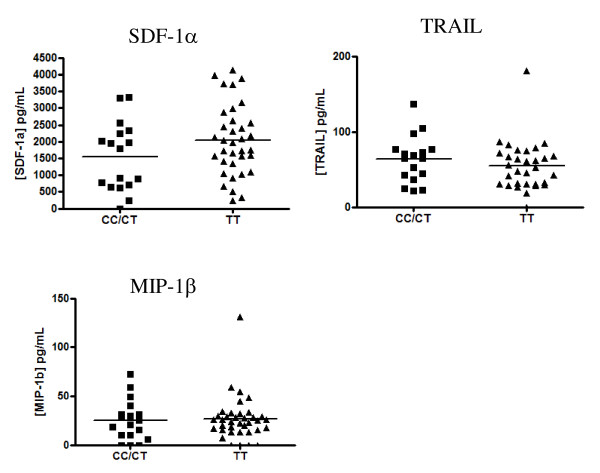
**Quantification of plasma cytokine/chemokine levels**. Plasma SDF-1α, MIP-1β and TRAIL concentrations were quantified by Milliplex bead assay among HIV-1-positive women of the CSW cohort. No significant differences in concentration between *GNB3 825CC/CT *and *825TT *groups were detected by Mann-Whitney test [p = 0.14 for SDF-1α, p = 0.88 for MIP-1β, p = 0.31 for TRAIL].

To complement the cell surface marker data, we assessed plasma concentrations of three cytokines/chemokines of interest: SDF-1α, MIP-1β and TRAIL. Given that 825TT patients are known to exhibit enhanced SDF-1α-mediated chemotaxis, we wondered whether plasma chemokine levels might vary between *GNB3 *genotype during infection. Additionally, some evidence points to lower levels of lymphocyte apoptosis in 825TT patients, leading us to assess plasma levels of the cleaved form of TNF-related apoptosis-inducing ligand (TRAIL, CD253). There were no significant differences in plasma TRAIL, SDF-1α or MIP-1β levels between CC/CT and TT genotypes [Mann-Whitney, p = 0.31, p = 0.14 and p = 0.88 respectively] (Figure 5). Subgroup analysis based on ARV treatment status did not reveal any further differences in protein concentration between genotypes (not shown).

## Discussion

This study assesses the impact of the *GNB3 825 *SNP on HIV-1 acquisition and disease progression in two Kenyan cohorts: a cohort of female commercial sex workers at high risk of sexual transmission of HIV (Pumwani CSW cohort), and a low-risk perinatal HIV transmission cohort (PHT cohort). Given the data indicating population-specific effects of the 825T allele and the high T allele frequency in African compared to Caucasian populations (80% versus 20%, respectively), it is important to understand the effect of the SNP in a population of high HIV-1 prevalence [9]. The effect of the SNP in the CSW cohort is of particular interest, as we have previously reported that a SNP in the *CD4 *gene, *CD4 C868T*, is associated with increased risk of HIV-1 acquisition in this cohort. The *CD4 *and *GNB3 *genes are located in close proximity on chromosome 12, and may contain SNPs in linkage disequilibrium. We, therefore, wondered whether the *GNB3 825T *allele might contribute to the epidemiological effects attributed to the *CD4 *SNP, but no significant linkage was found between the *GNB3 825T *and *CD4 868T *alleles.

Given recent evidence suggesting an important role for G protein signaling in promoting HIV-1 replication and entry into resting T cells, and the fact that the *GNB3 C825T *SNP is one of the most commonly described polymorphisms in human G protein subunit genes [1,3,23], it is important to attempt to replicate previously reported associations of the 825T allele with accelerated HIV-1 disease progression and HAART responses [7,8]. The association of the 825TT genotype with increased risk of HIV-1 progression, however, is somewhat at odds with other reports suggesting a protective effect of the T allele in infection. *GNB3 825T *is associated with improved cellular responses to Hepatitis B vaccination, and the 825CC genotype may confer a poor response to interferon α/ribavirin therapy in Hepatitis C infection as well as increased risk of infant death due to infection [19,24,25]. Given that epidemiological studies linking the 825T allele to non-infectious diseases have found evidence of population-specific effects with little functional data to demonstrate the mechanisms of action of the T allele, it is important to confirm the associations between *GNB3 825T *and HIV-1 progression, as well as to better understand the impact of the SNP on immune function during chronic disease.

Surprisingly, we were unable to demonstrate any effect of the *GNB3 825T *allele on HIV-1 acquisition or disease progression in either cohort. Although there was a trend toward increased risk of infant HIV-1 acquisition via breastmilk transmission (p = 0.06 adjusted for maternal viral load), this reflected a total of 16 transmission events (the least frequent mode of mother-to-child transmission observed in this cohort) and was not accompanied by a difference in maternal breastmilk viral load. The lack of association with either sexual or vertical HIV-1 transmission did not appear to be due to any confounding effect of the *CD4 868 *SNP. To date, there have been no reports of increased HIV-1 acquisition associated with GNB3 genotype in any population. Our analysis also failed to confirm the previously reported observation that the *GNB3 825TT *genotype is associated with increased HIV-1 disease progression as measured by CD4 decline [7]. Regardless of whether analysis was limited to patients followed from the time of seroconversion, or included HIV-1-positive participants with unknown seroconversion dates (with adjustment for baseline CD4 count), and whether disease progression endpoint was defined as CD4 < 350, CD4 < 250 and/or death, rate of CD4 decline or viral load increase over time, there was no association between GNB3 genotype and HIV-1 disease progression. A small subset of HIV-1-positive GNB3 genotyped patients from the CSW cohort for whom ARV initiation and CD4 count data were available were included in a survival analysis of time to CD4 rebound to > 300 cells/uL following ARV initiation. Among this group (n = 12 CC/CT, 17 TT), there was also no difference in response to ARV therapy (data not shown).

Given the high 825T allele frequency among African populations, it was important to determine whether *GNB3 *genotype would affect disease progression similarly to the reported effect in a study of Caucasian participants. The absence of a *GNB3 *effect on disease progression in this study does not necessarily imply that *GNB3 *genotype does not affect HIV progression in other ethnic populations; rather, it underscores the importance of replicating genetic association studies in populations with disparate allele frequencies. Furthermore, it cannot be ruled out that difference of cohort gender composition between this and previous studies contributed to the contrasting results. Studies of the *GNB3 825 *SNP in African populations are lacking in the literature, but our results, along with others [26], suggest that phenotypes associated with the 825T allele may not be detected in African populations, and reinforce the observation that more diverse studies are required. Another gap within the body of *GNB3 *literature lies in the lack of functional studies to accompany epidemiological analyses. Although the C to T mutation does not affect the amino acid sequence of the Gβ3 protein, it is associated with the production of truncated splice variants, Gβ3s and Gβ3s2 [5,17]. Biochemical characterization of these transcripts suggests that they are biologically active, and promote increased signaling activity following G protein coupled receptor activation. However, data demonstrating Gβ3s/s2 expression at the protein level *in vivo *are still lacking [5,27].

Given the documented effect of enhanced lymphocyte chemotaxis in healthy Caucasian 825T allele carriers [18] and the potential impact of the splice variants on immune signaling pathways, we assessed the impact of *GNB3 *genotype on cellular immune activation and plasma cytokine/chemokine levels in healthy and HIV-1-positive CSW cohort participants. No differences in acute or chronic activation marker expression (CD69, HLA-DR and CD38) on either CD4+ or CD8+ T cells were observed in HIV-1-negative participants, confirming and extending a previous report that HLA-DR expression was not altered across *GNB3 *genotypes in healthy Caucasians [20]. The same study also indicated that healthy Caucasian 825T allele carriers have increased CD4+ T cell counts (with no differences in B cell or CD8+ T cell counts), but did not assess the proportion of any CD4+ T cell subpopulations such as Tregs [20]. In the current study, we did not observe any differences in Treg proportion between *GNB3 *825CC/CT and TT participants.

Analyses among HIV-1-positive patients also failed to identify any differences in *ex vivo *immune activation as measured by CD69, HLA-DR, or CD38. To our knowledge, this is the first study to quantify activation markers among HIV-1-positive patients with respect to *GNB3 *genotype. We also measured expression of IL-7Rα (CD127) and Fas (CD95), two known correlates of disease progression. During HIV infection, IL-7Rα inversely correlates with immune activation and apoptosis, and positively correlates with CD4 count [22], while Fas expression positively correlates with disease progression [21,28]. Neither marker was differentially expressed on CD4+ or CD8+ T cells between GNB3 genotypes. Given the limitations of cross-sectional studies in assessing differences between markers correlated with disease progression, we analysed all surface marker expression with adjustment for CD4 count, but still observed no differences between *GNB3 *genotypes. *GNB3 825T *allele carriers demonstrate increased SDF-1α-mediated lymphocyte chemotaxis [18] (a process mediated by G protein signaling); therefore, we wondered whether CXCR4 ligand SDF-1α or CCR5 ligand MIP-1β would be differently expressed between *GNB3 *genotypes, particularly during HIV-1 infection. Plasma TRAIL levels were also assessed due to the previously reported observation of decreased lymphocyte apoptosis among 825TT individuals [14]. Consistent with the cell surface marker data, SDF-1α, MIP-1β and TRAIL levels were also similar between CC/CT and TT genotypes. While these results do not assess the impact of the *GNB3 *splice variants on intracellular signaling pathways nor imply that there is no impact of the 825 SNP on signal transduction, our data do suggest that *GNB3 *genotype does not have a substantial impact on co-receptor expression or the activation of bulk or CD4+CCR5+ T cells. Investigation of these markers in cohorts that do exhibit an impact of *GNB3 *genotype on HIV-1 progression may provide further information about the mediators of the rate of progression.

## Conclusions

Overall, our data indicate that the *GNB3 C825T *SNP does not play a significant role in HIV-1 acquisition, disease progression or immune activation in the African cohorts described. Given the population-specific effects of the *GNB3 825 *SNP, further data will be required to conclusively determine what role it may play in HIV-1 progression in non-African populations, and to more fully describe its mechanism of action.

## Methods

### Subjects

#### Pumwani Commercial Sex Worker Cohort

This retrospective study was performed in the Pumwani Commercial Sex Worker (CSW) cohort in Nairobi, Kenya which is an open cohort of female commercial sex workers who are highly exposed to HIV-1. The study was approved by the ethics review boards of both the Kenyatta National Hospital and the University of Manitoba. Participants in the cohort report for follow-up twice a year, when blood samples are taken for HIV-1 serology and CD4+ T cell count determination. *GNB3 *genotypes of 1031 participants were analysed for allele frequency and Hardy-Weinberg equilibrium, 969 of which were assessed for linkage disequilibrium with the *CD4 C868T *SNP. Analyses of time to HIV acquisition and disease progression were restricted to patients with complete follow-up data, resulting in a sample group of 204 HIV- and 219 HIV+ patients (73 of whom seroconverted during their follow-up in the cohort).

#### Perinatal HIV Transmission (PHT) Cohort

This cohort recruited HIV+ pregnant women from antenatal clinics in Nairobi, Kenya to investigate factors affecting *in utero*, postpartum and breastmilk HIV transmission. The study was approved by the ethics review boards of Kenyatta National Hospital, the University of Manitoba and the University of Washington. Participants were enrolled at 32 weeks gestation and began taking zidovudine twice a day from 34-46 weeks of pregnancy and following through delivery, as per Kenyan guidelines [29]. Women visited the clinic antenatally, at delivery, 2 weeks after birth and monthly for 12 to 24 months. Blood specimens were collected at months 1, 3, 6, 9, 12, 18 and 24 following delivery for CD4+ T cell count and HIV viral load (VL) determination. *GNB3 *genotype was determined for the 444 mothers and 395 children who were included in this study.

### DNA Isolation

CSW cohort DNA samples were isolated either from whole blood, peripheral blood mononucleocytes (PBMCs), or B cell lines using Qiagen DNA extraction kits or Qiagen BioRobot EZ1 (Qiagen, Inc., Mississauga, ON, Canada) following the manufacturer's instructions. DNA was isolated from PHT cohort maternal serum samples using the QiaAmp DNA Mini Kit serum protocol (Qiagen, Inc., Mississauga, ON, Canada), as described in the manufacturer protocol. DNA from infant bloodspots was isolated using the QiaAmp DNA Mini Kit dried bloodspot protocol as described.

### PCR and Sequence Analysis

Nested PCR amplification of the *GNB3 825T *locus was performed with the outer primer set (Table 2) at an annealing temperature of 60°C. 2 μl of the reaction was added as template for the nested reaction using the inner primer set (Table 2) at an annealing temperature of 60°C. Sequencing was performed in both directions using either the forward or reverse sequencing primers. Sequences were resolved on an ABI3100 sequencer, and genotyped by visual inspection using Sequencher and Codon Express software.

**Table 2:** Primers used in PCR amplification and sequencing of *GNB3 C825T *SNP

Primer Set	Forward Primer (5' - 3')	Reverse Primer (5'-3')	T_a _(^°^C)
Outer	GCTGCCCAGGTCTGATCCCT	CCAGTGACAAGGGACAGCAGTAAG	60

Inner	TGACCCACTTGCCACCCGTGC	GCAGCAGCCAGGGCTGGC	60

Sequencing	CAGTTCTTCCCCAATGGAGAGG	GGCTGGCCCTTACCCACACG	55

T_a_: annealing temperature.

### Viral Load Determination

HIV-1 RNA VL was quantified in plasma using the Gen-Probe Transcription Mediated Amplification assay, which is sensitive for detection of Kenyan HIV-1 subtypes A, C, and D (Gen-Probe Incorporated, San Diego, CA) [30].

### Multi-parametric Flow Cytometry

Peripheral blood mononuclear cells (PBMC) were isolated from fresh blood samples by layering onto ficoll-hypaque (Bio-Lynx). PBMC were immediately stained following isolation with flurochrome-conjugated antibodies specific to CD3, CD4, CD8, CD69, HLA-DR, CD38, IL-7Rα (CD127) and Fas (CD95) (BD Biosciences). Regulatory T cells were identified by expression of CD3, CD4, CD25 (BD Biosciences) and intracellular staining of FOXP3 (eBiosciences) according to manufacturer's instructions [31]. Data were collected on the BD LSRII using FACS Diva software, and analysed with FlowJo version 7.2.

### Plasma chemokine quantification

Plasma concentrations of SDF-1α, MIP-1β and TRAIL were quantified using the Milliplex Human Cytokine/Chemokine and Human Cytokine/Chemokine Panel II kits (Millipore) as per the manufacturer's short protocol. The assay was run on the BioRad Bio-Plex 200 and analysed with Bioplex Manager Software v5.0.

### Statistical Analysis

A total of 969 Pumwani CSWs were genotyped for both *CD4 868 *(rs28919570) and *GNB3 825 *(rs5443). The extent of linkage disequilibrium between these two SNPs (LOD score, D', and r^2^) was determined with Haploview version 4.2. Cox proportional hazard regression and Kaplan-Meier survival analysis were used to determine the association of *GNB3 *genotype with time to HIV acquisition and time to disease progression defined by time to CD4 < 350, CD4 < 250 and death. Linear mixed effect models were used to determine associations between *GNB3 *genotype and the rate of change in CD4 count and viral load over time. Linear regression was used to compare baseline CD4 count and log_10 _transformed plasma viral load among mothers. All analyses were specific *a priori *and data were analysed in SAS and SATA version 11 (College Station TX). Comparison of immune activation markers and plasma cytokine/chemokine concentrations was performed by non-parametric Mann-Whitney tests in GraphPad Prism version 4, and analysis of co-variance (ANCOVA) in SPSS version 16. P values were considered significant at p < 0.05.

## Competing interests

The authors declare that they have no competing interests.

## Authors' contributions

JJ contributed to genotyping, flow cytometry and statistical analysis, performed cytokine bead array assays and drafted the manuscript. JT contributed to genotyping and performed linkage analysis. RC contributed to statistical analysis. JK and CW were responsible for patient recruitment and sample collection. CC and SK contributed to flow cytometry assays. TB, CF, FP, GJS, ML and KF conceived of the study, participated in its design and coordination and helped to draft the manuscript. All authors read and approved the final manuscript.
